# How do carbohydrate quality indices influence on bone mass density in postmenopausal women? A case–control study

**DOI:** 10.1186/s12905-023-02188-4

**Published:** 2023-01-31

**Authors:** Mehran Nouri, Marzieh Mahmoodi, Zainab Shateri, Marzieh Ghadiri, Milad Rajabzadeh-Dehkordi, Mohebat Vali, Bahram Pourghassem Gargari

**Affiliations:** 1 Health Policy Research Center, Institute of Health, Shiraz University of Medical Sciences, Shiraz, Iran; 2 Student Research Committee, Shiraz University of Medical Sciences, Shiraz, Iran; 3 Department of Community Nutrition, School of Nutrition and Food Science, Shiraz University of Medical Sciences, Shiraz, Iran; 4 Nutrition Research Center, Shiraz University of Medical Sciences, Shiraz, Iran; 5 Student Research Committee, Ahvaz Jundishapur University of Medical Sciences, Ahvaz, Iran; 6 Student Research Committee, Department of Biochemistry and Diet Therapy, Faculty of Nutrition and Food Sciences, Tabriz University of Medical Sciences, Tabriz, Iran; 7 Nutrition Research Center, Department of Biochemistry and Diet Therapy, Faculty of Nutrition and Food Sciences, Tabriz University of Medical Sciences, Tabriz, Iran

**Keywords:** Carbohydrate quality index, Low-carbohydrate diet, Glycemic index, Glycemic load, Insulin load, Insulin index, Bone density

## Abstract

**Background:**

Carbohydrates are the primary energy source in Asian countries, including Iran. An emerging method can be used to measure the quality of carbohydrates, including the carbohydrate quality index (CQI), which includes a variety of components. Low-carbohydrate diet score (LCDS) has been proposed as a new method of scoring micronutrient intake that could provide a reasonable explanation for the link between diet and the risk of chronic diseases.

**Objective:**

This study aimed to investigate the relationship between CQI, LCDS, glycemic index (GI), glycemic load (GL), insulin load (IL), and insulin index (II) with bone mass density (BMD) in postmenopausal women.

**Method:**

In this case–control study, 131 postmenopausal women with osteoporosis/osteopenia and 131 healthy postmenopausal women aged 45–65 participated. The dual-energy X-ray absorptiometry (DEXA) method measured the BMD of the lumbar vertebrae and femoral neck. A validated semi-quantitative food frequency questionnaire was used to assess dietary intake. Logistic regression were used to evaluate the relation between GI, GL, II, IL, CQI, and LCDS with BMD.

**Results:**

Diets with higher GI increased the risk of osteopenia and osteoporosis, but LCDS and CQI decreased the risk of osteopenia and osteoporosis.

**Conclusion:**

These findings suggest that a higher intake of fruits and vegetables and receiving various dietary vitamins, minerals, and antioxidant compounds may be a useful way to prevent osteopenia in Iranian women.

## Introduction

Carbohydrates are the primary energy source in Asian countries, including Iran, due to the consumption of high amounts of potatoes, rice, and grains. It has been reported that increased carbohydrate consumption has adverse health effects because of increased energy intake and glycemic load (GL) response [1]. Carbohydrates are known to be the underlying cause of metabolic disorders, while less emphasis is considered on the carbohydrate source. In this line, higher consumption of whole grains, dietary fiber, and cereal fiber and a lower intake of refined grains and added sugar is associated with fewer disorders [2]. Therefore, as an indicator of carbohydrate quality, whole grains, and total dietary fiber have a more important role in determining health than the carbohydrate quantity [2–4].

Traditional methods were used to determine the quality of the carbohydrates, including the ratio of total carbohydrates to total dietary fiber (carb-to-fiber) or the ratio of total carbohydrates to cereal fiber (carb-to-cereal), which are associated with an increased risk of metabolic disorders [2]. However, an emerging method can be used to measure the quality of carbohydrates, including the carbohydrate quality index (CQI), which includes a variety of components such as dietary fiber, glycemic index (GI), whole plus refined grain, and solid to total carbohydrate ratio [2, 5]. Various studies have shown that a diet with higher CQI leads to higher-quality carbohydrate intake, which is associated with a reduced risk of metabolic disorders [2, 6–8].

As part of a dietary carbohydrate quality classification, the GI classifies the effects of various foods based on their carbohydrate absorption and glycemic response. A diet with a higher GI has been shown to include a higher intake of refined grains and a lower intake of whole grains and cereal fiber, which ultimately leads to an increased risk of various disorders and higher mortality [9]. Also, Shahdadian et al. have reported an association between GI and mortality in women [10]. These effects are associated with elevated glucose and insulin levels in response to higher GI diets. Because higher glucose levels induce oxidative stress and inflammation, decrease osteoblast activity, and increase bone resorption due to the induction of acidosis, adversely affecting bone health [11].

Evidence has shown that a high-carbohydrate diet or more consumption of white rice with a low-fat diet plays an essential role in the development and management of various disorders in Asian countries [1, 12]. In this view, the effects of micronutrient intake on dietary patterns play an important role in assessing the relationship between diet and disease [1]. Low-carbohydrate diet score (LCDS) has been proposed as a new method of scoring micronutrient intake that could provide a reasonable explanation for the link between diet and the risk of chronic diseases [1, 13, 14]. LCDS is associated with lower carbohydrate intake and higher fat and protein intake and defines the ratio of all macronutrients in a dietary pattern [1].

Since no study has been done in this field so far, the purpose of this study was to investigate the relationship between CQI, LCDS, GI, insulin load (IL), and insulin index (II) with bone mass density (BMD) in postmenopausal women.

## Methods

### Study population

**Fig. 1:**
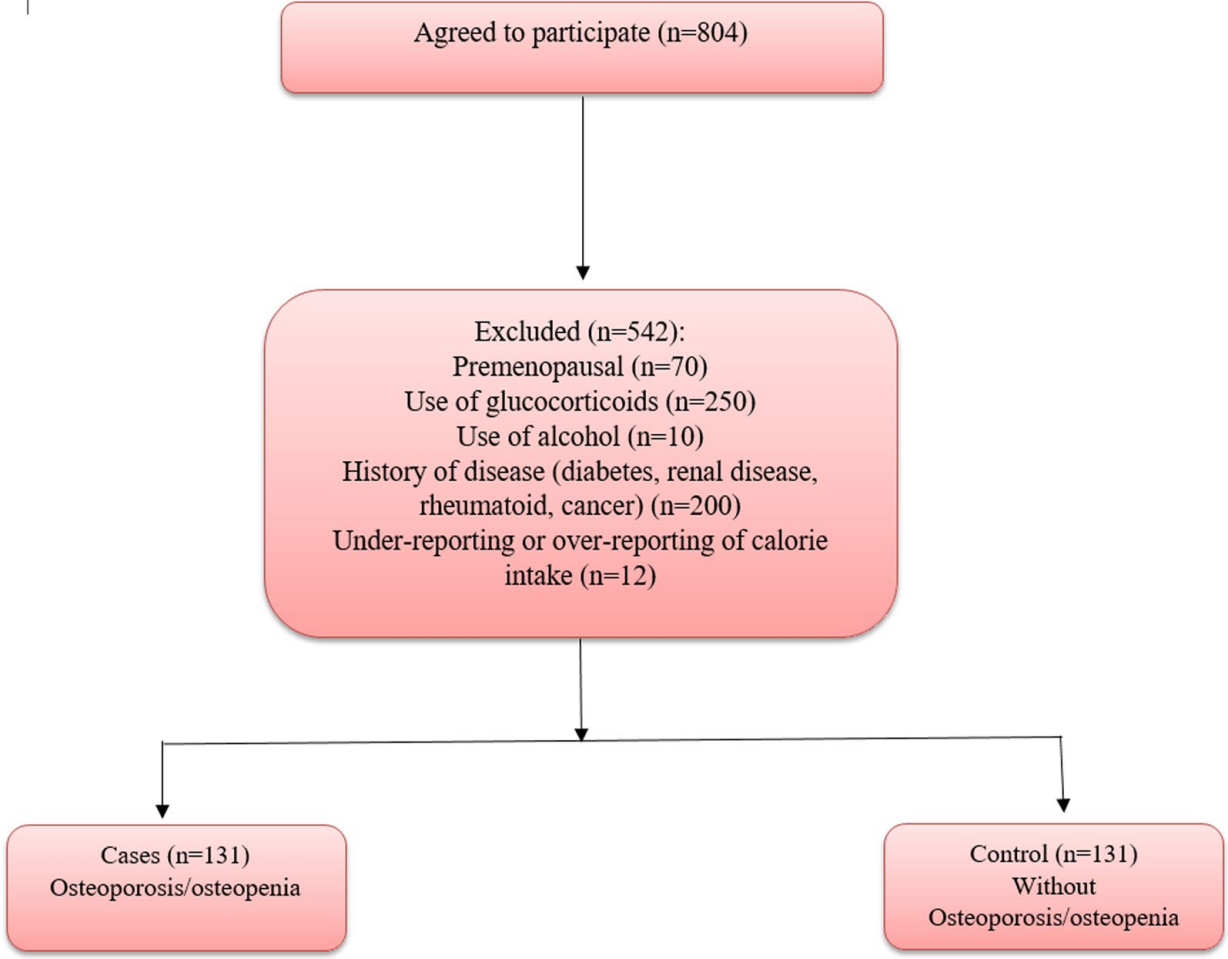
Flow chart of the study

In this case–control study, 131 postmenopausal women with osteoporosis/osteopenia without fracture and 131 healthy postmenopausal women aged 45–65 participated. These women were selected from the Bone Densitometry Center in Isfahan city, Iran, from May 2021 to December 2021. The sample size was computed based on the previous research, considering OR = 2.30 [15]. Menopause was determined as the absence of a menstrual cycle in the last twelve months. The study exclusion criteria included premenopausal, alcohol and glucocorticoid consumption, diabetes, rheumatoid, cancer, renal disease, and history of chemotherapy (Fig. 1). Some detailed of the study have been published previously [16].

Participants' body weight was measured with a digital scale and noted with a precision of 100 g. A stadiometer measured height with an accuracy of 0.5 cm, and then body mass index (BMI) was determined. Some information, such as sociodemographic variables, smoking, and use of drugs or supplements, was gathered by a general information questionnaire.

The dual-energy X-ray absorptiometry (DEXA) method measured the BMD of the lumbar vertebrae and femoral neck (the device model was Horizon Wi (S/N 200451)). The status of bone mass was assessed by the World Health Organization (WHO) criteria (T score greater than − 1 means normal bone mass, T score between − 1 and − 2.5 means osteopenia, and T score less than − 2.5 means osteoporosis) [17]. If a physician identified osteoporosis and osteopenia, they were selected for the case samples, and their controls were selected without osteoporosis.

The physical activity level was gathered by the international physical activity questionnaire (IPAQ) [18]. According to metabolic equivalent of task [3]-minutes and standard protocol, women were placed into three groups (less than 600 metabolic equivalent of task (MET)-minutes/week: low activity, 600–3000 MET-minutes/week: moderate activity, and more than 3000 MET-minutes/week: intense activity).

### Dietary assessment and food grouping

Participants completed a validated food frequency questionnaire (FFQ) [19]. Baseline dietary intake data which was taken from food composition tables, was used to recognize CQI according to the four criteria: the GI, the ratio of carbohydrates of whole grains to carbohydrates of total grains, the proportion of solid carbohydrates to total carbohydrates, and total dietary fiber intake. Liquid carbohydrates such as fruit juice and sugar-sweetened beverage consumption, while solid carbohydrates were matched to the carbohydrate content of the rest of the meal with each carbohydrate content. The total score range was between 4 and 20 (higher amounts mean better quality of carbohydrates) [7, 20].

For calculating the LCDS, all participants were divided into 11 strata for carbohydrate, refined grains, monounsaturated fatty acid (MUFA), vegetable protein intake, fiber (g/1000 kcal), GL, and polyunsaturated fatty acid (PUFA)(n3/n6) [14]. For MUFA, n3/n6 PUFA, fiber, and vegetable protein, participants in the highest stratum got 10 points and lowest stratum got 0 points. For refined grains, GL and carbohydrates, the intake of the lowest carbohydrate got 10 points and the intake of the highest carbohydrate got 0 points. Finally, the overall diet score was between 0 (the lowest intake of protein and fat and the highest intake of carbohydrates) and 70 (the highest intake of protein and fat and the lowest carbohydrates). So the higher the score, the higher the low-carbohydrate diet pattern named “LCDS”.

GI was calculated from this formula: (GI × available carbohydrate)/total available carbohydrate. Available carbohydrate means total carbohydrate minus fiber [21]. The United States Department of Agriculture food composition table was used for total carbohydrate and fiber content. Iranian GI table was just used for 6 out of 85 foods [21]. For 62 other foods, international tables were used [21], and for 17 foods, we used similar foods because the GI of these foods was not accessible. The GL was calculated from this formula: (total GI × total available carbohydrate/100).

II refers to the increase in insulin level under the curve during 2 h in reaction to 1000 kJ of test food divided by the area under the curve after consumption of 1000 kJ of reference food. Previous studies were used to obtain the II. II of similar foods was used for some items that were not in the list of foods based on the relationship between carbohydrate, fiber, protein, fat, and energy content. For example, raisins were used for dates. To assess IL for each person, IL was calculated (II of the food × energy of one gram of that food × amount of the food eaten) then the IL of each food was summed and then II was calculated (IL/total energy intake) [22–25].

### Ethics statement

The protocols of this study were confirmed by the Ethical Committee of Tabriz University of Medical Sciences (IR.TBZMED.REC.1400.114), and all individuals completed written consents.

### Statistical analysis

We used SPSS (version 24.0, SPSS Inc., Chicago IL, USA) for statistical analysis. Also, figure depicted by R software version 3.0.2. A p-value less than 0.05 was considered statistically significant. For evaluating the normal distribution of data, the Kolmogorov–Smirnov test was used. Means and standard deviations (SDs) were used for the continuous variables, and frequencies or percentages were used for the categorical variables. We used an independent samples T-test to analyze of nutrients and food items intake between the case and control.

Moreover, the crude and two adjusted model logistic regression were used to evaluate the relation between BMD and CQI. The effects of BMI and age were controlled in the first adjusted model. In the second model, the role of income, education, physical activity, calcium, and vitamin D supplements were also adjusted.

## Results

The baseline characteristics of the participants are presented in Table 1. The age (P = 0.03), the BMD femoral and lumbar (P < 0.001 for both), physical activity level (P = 0.01), education level (P < 0.001), and vitamin D use (P = 0.01) was different between the case and control groups.

**Table 1:** Baseline characteristics of study participants

Variables	Control (n = 131)	Case (n = 131)	P-value*
Age (year)	56.47 ± 5.91	57.95 ± 5.42	**0.03**
BMI (kg/m^2^)	29.13 ± 3.31	29.78 ± 3.99	0.15
BMD femoral	0.78 ± 0.07	0.64 ± 0.09	**< 0.001**
BMD lumbar	1.00 ± 0.08	0.81 ± 0.09	**< 0.001**
Income, average (%)	53 (40.5)	65 (49.6)	0.08
Physical activity, low (%)	109 (83.2)	122 (93.1)	**0.01**
Education level (%)			**< 0.001**
Under diploma	65 (49.6)	98 (74.8)	
Diploma	52 (39.7)	25 (19.1)	
Higher diploma	14 910.7)	8 (6.1)	
Calcium Supplement, no (%)	99 (75.6)	99 (75.6)	0.55
Vitamin D Supplement, no (%)	55 (42.0)	73 (55.7)	**0.01**

Values are shown as mean (SD) for continuous and percentage for categorical variables

*BMI* body mass index, *BMD* bone mineral density

*Using independent samples T-test for continuous and chi-square test for categorical variables

P-values marked with bold indicate statistically significant p-values

Based on Table 2, the intake of protein (P = 0.001), fiber (P ˂ 0.001), vitamin A (P = 0.003), K (P = 0.001), B_2_ (P = 0.005), B_3_ (P = 0.001), B_6_ (P ˂ 0.001), B_9_ (P = 0.01), C (P ˂ 0.001), beta-carotene (P ˂ 0.001), potassium (P = 0.001), iron (P = 0.03), calcium (P = 0.008), magnesium (P = 0.004), zinc (P = 0.04) and copper (P = 0.01) was higher, but PUFA (P = 0.02), omega 6/3 ratio (P = 0.03), and sodium (P = 0.001) was lower in the control group compared to the case group.

**Table 2:** Nutrient intakes between study participants

Variables	Control (n = 131)	Case (n = 131)	P-value*
Energy (kcal/d)	2121.10 ± 357.82	2132.82 ± 361.94	0.79
Protein (g/day)	69.77 ± 13.19	64.63 ± 12.33	**0.001**
Carbohydrate (g/day)	314.36 ± 50.74	314.49 ± 54.58	0.98
Fat (g/day)	72.72 ± 14.03	75.57 ± 14.67	0.11
Fiber (g/day)	32.59 ± 5.98	29.57 ± 5.81	**< 0.001**
Cholesterol (mg/day)	155.65 ± 46.42	151.12 ± 48.20	0.44
SFA (g/day)	18.76 ± 4.71	18.68 ± 5.13	0.89
MUFA (g/day)	26.42 ± 4.97	27.07 ± 4.59	0.27
PUFA (g/day)	18.45 ± 3.99	19.49 ± 3.24	**0.02**
Omega 6/3 ratio	22.97 ± 10.27	27.25 ± 12.52	**0.03**
Vitamin A (RAE/day)	535.15 ± 278.92	434.77 ± 255.95	**0.003**
Beta-carotene (mg/day)	4268.77 ± 2794.49	3113.86 ± 2330.65	< **0.001**
Vitamin E (mg/day)	21.60 ± 4.84	22.60 ± 4.15	0.07
Vitamin K (mg/day)	145.56 ± 82.41	114.48 ± 64.70	**0.001**
Vitamin B_1_ (mg/day)	1.86 ± 0.29	1.80 ± 0.26	0.09
Vitamin B_2_ (mg/day)	1.72 ± 0.48	1.56 ± 0.45	**0.005**
Vitamin B_3_ (mg/day)	20.43 ± 3.13	19.22 ± 2.72	**0.001**
Vitamin B_6_ (mg/day)	1.78 ± 0.36	1.60 ± 0.32	< **0.001**
Vitamin B_9_ (µg/day)	476.47 ± 83.27	451.35 ± 75.72	**0.01**
Vitamin B_12_ (µg/day)	2.98 ± 1.34	2.79 ± 1.50	0.28
Vitamin C (mg/day)	160.03 ± 71.32	125.97 ± 65.09	< **0.001**
Sodium (mg/day)	3573.28 ± 507.61	3797.38 ± 537.79	**0.001**
Potassium (mg/day)	3341.60 ± 825.11	2996.10 ± 808.42	**0.001**
Iron (mg/day)	15.45 ± 2.19	14.88 ± 2.12	**0.03**
Calcium (mg/day)	538.17 ± 306.33	439.93 ± 286.41	**0.008**
Magnesium (mg/day)	429.60 ± 71.95	403.21 ± 74.32	**0.004**
Zinc (mg/day)	11.30 ± 2.22	10.72 ± 2.38	**0.04**
Copper (mg/day)	1.62 ± 0.27	1.53 ± 0.28	**0.01**

Values are shown as mean (SD)

*SFA* saturated fatty acid, *PUFA* polyunsaturated fatty acid, *MUFA* monounsaturated fatty acid, *RAE* retinol activity equivalent

*Using independent samples T-test

P-values marked with bold indicate statistically significant p-values

**Fig. 2:**
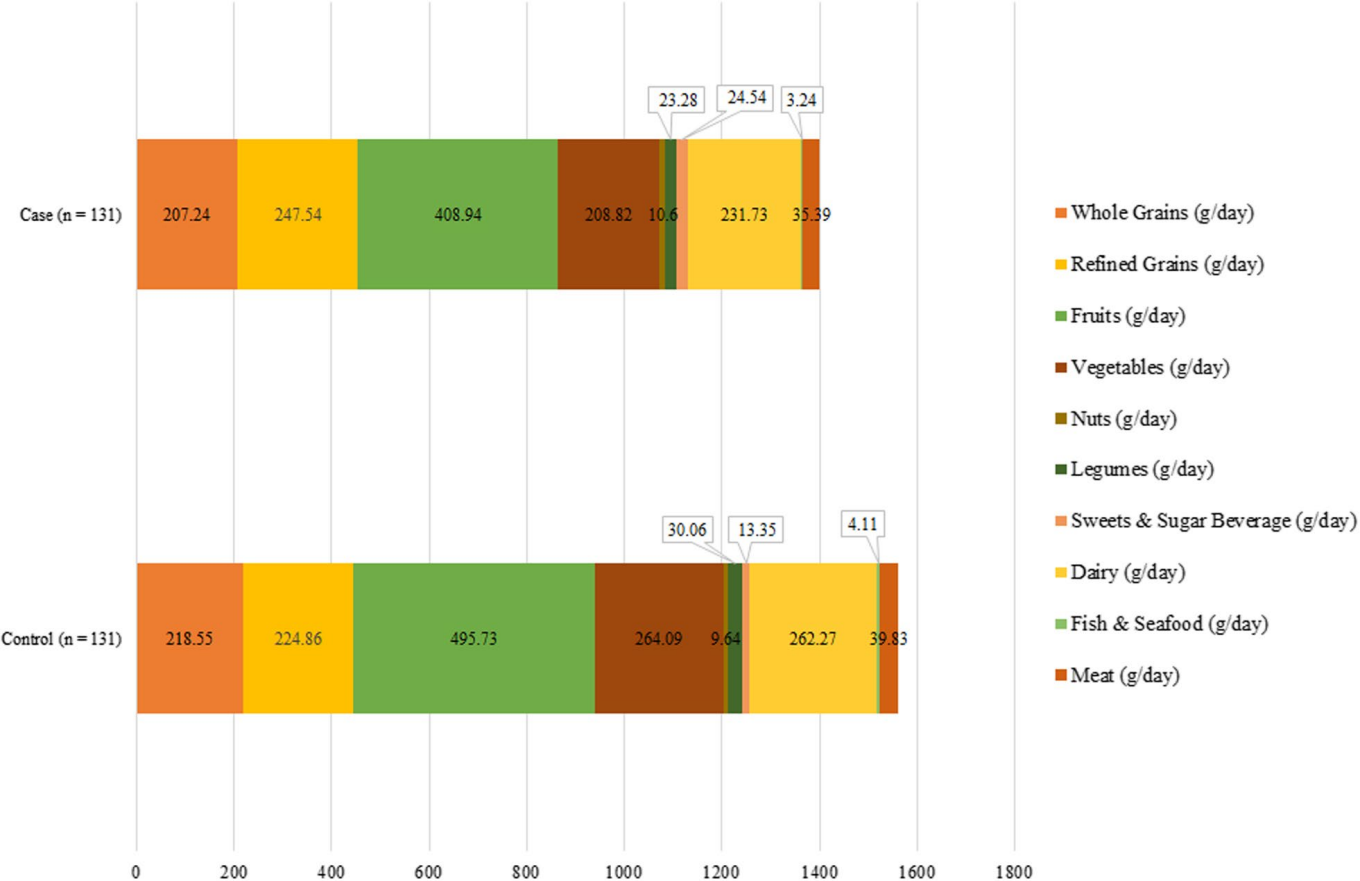
Food group intakes between study participants

According to Fig. 2, the intake of fruits (P = 0.001), vegetables (P ˂ 0.001), legumes (P ˂ 0.001), and meat (P = 0.008) was higher, but sweets and sugar beverages (P = 0.01), was lower in the control group compared to case group.

Table 3 presented the multivariable-adjusted odds ratios and 95% confidence intervals (ORs; 95% CI) for the femoral and lumbar bone density across tertiles (T) of GI, GL, II, IL, CQI, and LCDS. Individuals in the second and last tertile of GI were more likely to have lower femoral density in the crude model (T_2_-OR 2.55; 95% CI 1.38–4.64 and T_3_-OR 2.16; 95% CI 1.18–3.97) and both adjusted models (Model 1: T_2_-OR 2.49; 95% CI 1.34–4.64 and T_3_-OR 2.22; 95% CI 1.20–4.13; and Model 2: T_2_-OR 2.59; 95% CI 1.35–4.96 and T_3_-OR 2.02; 95% CI 1.06–3.87) compared those in the lowest tertile. Also, a positive association was seen between the second and last tertile of GI with the lumbar density in the crude model (T_2_-OR 2.26; 95% CI 1.23–4.14 and T_3_-OR 3.10; 95% CI 1.67–5.67) and both adjusted models (Model 1: T_2-_OR 2.21; 95% CI 1.20–4.08 and T_3_-OR 3.18; 95% CI 1.70–5.93; and Model 2: T_2-_OR 2.13; 95% CI: 1.13–4.01 and T_3_-OR 3.06; 95% CI 1.60–5.84).

**Table 3:** Crude and multivariable-adjusted odds ratios and 95% CIs across tertile of GI, GL, II, IL, CQI and LCDS

Variables	Femoral BMD abnormality			Lumbar BMD abnormality		
	Crude	Adjusted model 1	Adjusted model 2	Crude	Adjusted model 1	Adjusted model 2
*GI*						
T1	Ref	Ref	Ref	Ref	Ref	Ref
T2	2.55 (1.38, 4.69)	2.49 (1.34, 4.64)	2.59 (1.35, 4.96)	2.26 (1.23, 4.14)	2.21 (1.20, 4.08)	2.13 (1.13, 4.01)
T3	2.16 (1.18, 3.97)	2.22 (1.20, 4.13)	2.02 (1.06, 3.87)	3.10 (1.67, 5.76)	3.18 (1.70, 5.93)	3.06 (1.60, 5.84)
P_trend_	**0.01**	**0.01**	**0.02**	**˂ 0.001**	**˂ 0.001**	**0.001**
*GL*						
T1	Ref	Ref	Ref	Ref	Ref	Ref
T2	1.04 (0.57, 1.91)	1.09 (0.58, 2.03)	1.03 (0.53, 1.98)	1.28 (0.71, 2.32)	1.38 (0.74, 2.54)	1.26 (0.66, 2.40)
T3	0.79 (0.43, 1.44)	0.77 (0.41, 1.46)	0.82 (0.42, 1.61)	1.31 (0.72, 2.39)	1.40 (0.75, 2.62)	1.47 (0.76, 2.84)
P_trend_	0.44	0.42	0.55	0.36	0.29	0.27
*II*						
T1	Ref	Ref	Ref	Ref	Ref	Ref
T2	1.02 (0.56, 1.85)	0.97 (0.53, 1.79)	0.87 (0.46, 1.66)	1.38 (0.76, 2.51)	1.36 (0.74, 2.47)	1.25 (0.67, 2.33)
T3	1.02 (0.56, 1.86)	1.06 (0.57, 1.95)	0.93 (0.48, 1.77)	1.25 (0.69, 2.28)	1.30 (0.71, 2.38)	1.16 (0.62, 2.18)
P_trend_	0.94	0.85	0.86	0.44	0.38	0.58
*IL*						
T1	Ref	Ref	Ref	Ref	Ref	Ref
T2	0.79 (0.43, 1.44)	0.85 (0.46, 1.58)	0.85 (0.44, 1.63)	1.00 (0.55, 1.81)	1.07 (0.58, 1.96)	1.08 (0.57, 2.04)
T3	0.81 (0.44, 1.48)	0.83 (0.44, 1.55)	0.91 (0.47, 1.77)	1.00 (0.55, 1.82)	1.05 (0.56, 1.95)	1.17 (0.61, 2.24)
P_trend_	0.49	0.56	0.72	0.99	0.87	0.73
*CQI*						
T1	Ref	Ref	Ref	Ref	Ref	Ref
T2	0.74 (0.40, 1.34)	0.72 (0.39, 1.33)	0.73 (0.38, 1.37)	0.34 (0.18, 0.63)	0.33 (0.18, 0.62)	0.30 (0.15, 0.58)
T3	0.54 (0.29, 0.99)	0.49 (0.26, 0.91)	0.53 (0.28, 1.02)	0.28 (0.15, 0.52)	0.26 (0.13, 0.49)	0.26 (0.13, 0.50)
P_trend_	**0.04**	**0.02**	0.06	**˂ 0.001**	**˂ 0.001**	**˂ 0.001**
*LCDS*						
T1	Ref	Ref	Ref	Ref	Ref	Ref
T2	0.90 (0.49, 1.66)	0.80 (0.42, 1.49)	0.69 (0.36, 1.33)	0.74 (0.40, 1.37)	0.66 (0.35, 1.24)	0.57 (0.30, 1.11)
T3	0.78 (0.43, 1.42)	0.68 (0.36, 1.27)	0.70 (0.36, 1.34)	0.42 (0.23, 0.77)	0.37 (0.19, 0.69)	0.36 (0.19, 0.70)
P_trend_	0.42	0.23	0.29	**0.005**	**0.002**	**0.003**

*BMD* bone mass density, *GI* glycemic index, *GL* glycemic load, *II* insulin index, *IL* insulin load, *CQI* carbohydrate quality index, *LCDS* low-carbohydrate diet score

Model 1: adjusted for BMI and age

Model 2: additionally adjusted for income, education, physical activity, calcium, and vitamin D supplement

These values are shown as odds ratio (95% CIs)

Obtained from logistic regression

P-values marked with bold indicate statistically significant p-values

Moreover, we observed a significant negative relationship between CQI with the femoral BMD in the crude (OR 0.54; 95% CI 0.29–0.99) and the first adjusted model (OR 0.49; 95% CI 0.26–0.91). However, for the lumbar BMD, the association was significant in the second and last tertile of CQI compared to the first tertile in the crude model (T_2_-OR 0.34; 95% CI 0.18–0.63 and T_3_-OR 0.28; 95% CI 0.15–0.52) and both adjusted models (Model 1: T_2_-OR 0.33; 95% CI 0.18–0.62 and T_3_-OR 0.26; 95% CI 0.13–0.49; and Model 2: T_2_-OR 030; 95% CI 0.15–0.58 and T_3_-OR 0.26; 95% CI 0.13–0.50). Also, we observed a negative relationship between the last tertile of LCDS with the lumbar BMD in the crude model (OR 0.42; 95% CI 0.23–0.77) and both adjusted models (Model 1-OR 0.37; 95% CI 0.19–0.69; Model 2-OR 0.36; 95% CI 0.19–0.70) compared with those on the lowest tertile.

## Discussion

In this case–control study, our results showed that postmenopausal women with osteoporosis/osteopenia in Iran received less fruits, vegetables, vitamins and minerals. A higher GI intake and subsequently a lower CQI diet was also demonstrated by women with osteoporosis/osteopenia.

As a major energy source, carbohydrates play an essential role in bone metabolism. In this regard, the amount of carbohydrate and the quality of carbohydrate are considered important factors [26]. The dietary GI was presented by Jenkins et al*.* in 1981. This index was defined as glucose available after food digestion, which was used to measure carbohydrate quality [27].

The GI, as a component of CQI, affects glucose fluctuations. Therefore, CQI was associated with insulin resistance. Consumption of foods with high GI by reducing blood glucose fluctuations leads to enhanced appetite and energy intake. However, a low GI diet declined appetite and energy intake by increasing fluctuations in blood glucose [5]. This view demonstrated that eating foods with a high GI increase fat stores by reducing fat oxidation and increasing carbohydrate oxidation [5, 28, 29]. Hence, it has been shown that increasing fat stores by releasing pro-inflammatory cytokines, free fatty acids, and reactive oxygen species leads to inflammatory conditions that contribute to insulin resistance [30]. In addition, high GI sweetened beverages are associated with increased blood glucose levels and insulin resistance [5], and through fructose, they help increase fat storage by stimulating lipogenesis, increasing triacylglycerol accumulation, fat hypertrophy, and eventually insulin resistance [1]. Elevated blood glucose following the consumption of foods with high GI causes an increased need for insulin secretion and impaired beta cell function and glucose metabolism [1, 31]. Hence, enhanced glucose levels with a negative effect on osteoblast and osteoclast function, including overstimulation of the insulin signaling pathway and inhibitory effect on osteoblast cells, lead to abnormal bone metabolism and subsequent increase in fractures [11, 32, 33]. By stimulating the production of advanced glycation end products [34], hyperglycemia leads to increased cross-link between collagen, bone fractures, cell apoptosis, and inflammation [11]. In contrast, the consumption of whole grains with low GI reduces blood glucose levels and insulin resistance by reducing digestion and starch absorption [5, 35, 36]. Therefore, it seems that a low GI diet can reverse hyperglycemia, insulin resistance, inflammation, and oxidative stress, followed by bone health explains the findings of the study by Garcia-Gavilan et al. [11] which is consistent with our findings.

In the present study, the quantity of carbohydrates by determining LCDS was considered in addition to the quality of carbohydrates. A LCDS is associated with a reduction in carbohydrate intake and an increase in fat and protein intake. Higher LCDS have been reported to be associated with lower carbohydrate intake obtained from whole grains, fruits, and vegetables, and reduced intake of simple sugars, especially fructose [1]. In the Iranian population, a decrease in fruit and vegetable intake and an increase in simple sugar intake have also been reported [5, 37]. It has also been shown that in Iranians, more than 60% of total energy is obtained from carbohydrates, especially refined carbohydrates with a high GI [5]. Hence, higher LCD scores have been suggested to have beneficial health effects by replacing low GI fruits and vegetables, whole grains, and healthy sources of fat and protein with high GI refined carbohydrates [38]. These findings explain our results about how the higher LCDS associated with the higher BMD and thereby prevents osteopenia.

Observational studies have shown that a higher intake of fruits and vegetables is associated with increased BMD and decreased bone loss and fracture rate [39–41]. Lin et al*.* reported that receiving the Dietary Approaches to Stop Hypertension (DASH) diet for three months was associated with an increased intake of fruits and vegetables, significantly reduced bone turnover markers in 76-year-old men and women [42]. Our findings are consistent with these results and support the hypothesis that a higher intake of fruits and vegetables may be benefit for bone health. Mechanisms related to the effect of fruits and vegetables on bone health include the following:

First, providing a favorable ratio of sodium to potassium intake and reducing the diet's acidity, and thus reducing hypercalciuria [43]. The acid–base hypothesis has been reported that bone tissue buffered acid loading, leading to bone resorption and reduced bone density [43]. Because of their richness in alkaline ions such as potassium, calcium, and magnesium, fruits and vegetables cause alkaline conditions. It has been shown that all three elements have a beneficial effect on bone health [39]. A recent meta-analysis has shown that the richness of fruits and vegetables from these alkaline precursors counteracts the effects of dietary-derived calciuria [44, 45]. From this perspective, a meta-analysis of 17 studies reported that adequate calcium intake of 1200 mg/day is associated with a reduction in BMD loss in postmenopausal women, followed by a reduced fracture risk [46, 47].

In contrast, severe dietary calcium restriction leads to severe hypercalciuria and progressive loss of BMD [46]. Regarding the effect of magnesium on bone health, it has been shown that it leads to a significant increase in osteoblast survival, alkaline phosphatase activity, and osteocalcin levels [46, 48]. Therefore, insufficient magnesium intake is associated with osteopenia [46].

Second, they are rich in vitamin K, which plays a crucial role in bone health due to the gamma-carboxylation of osteocalcin [39, 44, 49]. Vitamin K plays an important role as a cofactor of enzymes involved in bone metabolism by increasing many bone formation markers such as alkaline phosphatase and insulin-like growth factor 1 (IGF-1) through osteoblast differentiation and by regulating extracellular matrix mineralization [46, 50–52]. The role of vitamin K in bone health has been reported in various studies [46, 53, 54].

Third, receiving antioxidants such as vitamin C, beta-carotene, and other carotenoids such as lutein, xanthine, and lycopene, which due to their antioxidant properties, have a protective role against oxidative stress [39]. In animal models, the protective effect of beta-carotene against bone loss has also been shown [46, 55]. Vitamin C may affect bone mass through the hydroxylation of proline and lysine, which is required to form triple helix collagen [44]. In addition, vitamin C has a protective effect against bone fractures by stimulating collagen synthesis types 1 and 3, while vitamin C deficiency leads to osteoclastogenesis and subsequent bone resorption. Adequate vitamin C intake and improved bone health can be a preventive tool against osteopenia and osteoporosis [46].

Fourth, receiving polyphenols, carotenoids, tocopherols, vitamin K, and glutathione, except for the antioxidant effects due to up-regulating Runt-related transcription factor 2 (Runx2), Osterix, and IGF-1 along with increasing the expression of lysyl oxidase, have beneficial effects on bone health [39].

Fifth, being rich in B vitamins can have a desirable effect on bone health. Adequate vitamin B_6_ intake has a positive effect on bone health, directly affecting bone metabolism and indirectly modulating steroid hormone receptors such as estrogen [46, 56]. The role of B_9_ in maintaining bone health has also been shown [46].

This study is the first one that assessed the relationship between carbohydrate quality indices and BMD in postmenopausal women. Also, we controlled the effect of several confounding factors in order to obtain more accurate results. However, this case–control study had some limitations: selection and recall bias that affect association is probable for the design of the study. Misclassification of the dietary intake of participants due to the use of FFQ cannot be omitted; however, we used validated FFQ, and finally, we were not capable of doing stratified analysis for a limited sample size.

In conclusion, higher GI diets increased the risk, but LCD and CQI decreased the risk of osteopenia and osteoporosis. These findings suggest that a higher intake of fruits and vegetables and receiving various dietary vitamins, minerals, and antioxidant compounds may be a useful way to prevent osteopenia and osteoporosis in Iranian women. Also, further studies with a longitudinal design, particularly trial studies and a higher sample size, are required to explain the better association between CQI and osteoporosis risk among this population.

## Data Availability

Data are available through a reasonable request from the corresponding author.
